# Combination of intracostal sutures with muscle flap to decrease post thoracotomy pain: A single blinded randomized clinical trial

**DOI:** 10.12669/pjms.331.11581

**Published:** 2017

**Authors:** Majid Montazer, Shahryar Hashemzade, Reza Movassaghi Gargari, Ali Ramouz, Sarvin Sanaie, Seyed Ziaeddin Rasihashemi

**Affiliations:** 1 Majid Montazer, Assistant Professor, Dept. of Cardiothoracic Surgery, Imam Reza Hospital, Tabriz University of Medical Sciences, Tabriz, Iran; 2 Shahryar Hashemzade, Professor, Dept. of Cardiothoracic Surgery, Imam Reza Hospital, Tabriz University of Medical Sciences, Tabriz, Iran; 3 Reza Movassaghi Gargari, Assistant Professor, Dept. of Anesthesiology, Faculty of Medicine, Tabriz University of Medical Sciences, Tabriz, Iran; 4 Ali Ramouz, Research Fellow, Dept. of Cardiothoracic Surgery, Faculty of Medicine, Tabriz University of Medical Sciences, Tabriz, Iran; 5 Sarvin Sanaie, Assistant Professor, Tuberculosis and Lung Disease Research Center, Tabriz University of Medical Sciences, Tabriz, Iran; 6 Seyed Ziaeddin Rasihashemi, Assistant Professor, Dept. of Cardiothoracic Surgery, Imam Reza Hospital, Tabriz University of Medical Sciences, Tabriz, Iran

**Keywords:** Intercostal muscle flap, Postoperative pain, Thoracotomy, Intracostal sutures

## Abstract

**Objective:**

To assess the efficacy of intercostal nerve protection by intercostal muscle (ICM) flap in post-thoracotomy pain improvement compared to intracostal suturing.

**Methods:**

In a randomized controlled trial, ninety-four patients undergoing posterolateral thoracotomy surgery were divided into two subgroups. Intracostal sutures in isolation and in combination with ICM flap techniques were used for thoracotomy closure in both groups. Numeric Pain Scale and Visual Pain Scale as pain scores were assessed on the first, second, third, fourth, fifth, sixth and seventh postoperative days and follow-up visits during the 2^nd^ week, 1^st^, 2^nd^, 4^th^ and 6^th^ months after thoracotomy.

**Results:**

Out of 94 patients, 58 were male and 36 were females. While the mean age of patients in intracostal group was 45.3 ± 17.6 years, it was 47.4 ± 16.1 years in intracostal plus ICM flap group. The mean operation time for the first group was 191.0 ± 74.7 minutes, while it was 219.3 ± 68.8 minutes in the second (p>0.05). Numeric rating score and visual pain scale did not demonstrate any significant difference in pain severity on postoperative days and follow-up visits between both groups (p>0.05). Although the trend of pain reduction was significant in each group (p<0.001), the difference was not statistically significant (p>0.001).

**Conclusion:**

Intracostal sutures in combination with muscle flap did not reduce postoperative pain in thoracotomy compared with intracostal sutures alone in thoracotomy closure.

## INTRODUCTION

Posterolateral thoracotomy is a widely performed procedure accompanied with post-operative pain and numerous studies have suggested intraoperative factors to influence the prevalence of this complication.^1^-^3^ Due to the several reports known about the underlying cause of the chronic post thoracotomy pain, intercostal nerve damage has been suggested to be the most common reason.^4^,^5^ Therefore, in order to decrease the post-thoracotomy pain, intraoperative protection of intercostal nerves should be taken into consideration.^6^,^7^ Intercostal nerve preservation could be performed by intracostal suture, or intercostal muscle flap techniques in order to decrease pain during thoracotomy.^8^,^9^

Despite several studies, chronic pain after thoracotomy is still causing significant problems influencing postoperative life quality of patients. Although, there are some powerful meta-analysis and controlled trials,^9^,^10^ evidences do not sufficiently persuade thoracic surgeons to employ an individual closure technique. Therefore, with due attention to diverse opinions on efficacy of intercostal muscle flap for intercostal nerve preservation compared to intracostal suture only, the current study was designed to compare the intensity of post-operative pain in asymptomatic patients that underwent posterolateral thoracotomy with and without intercostal muscle flap.

## METHODS

After obtaining informed consent all patients admitted for posterolateral thoracotomy in cardiothoracic department of Tabriz Imam Reza Hospital from March 2012 to March 2015, were enrolled for this study. (IRCT NO: 2015122216077N4) The exclusion criteria were: presence of any pain before surgery, long-term use of non-steroidal anti-inflammatory drugs (NSAIDs) or analgesics, narcotic addiction, psychiatric disease, radiologic evidence of invasion to parietal pleura or rib, age lower than 19, history of receiving corticosteroid in last six months, preoperative chemotherapy or radiotherapy, neuropathy, previous thoracotomy, epidural catheter dysfunction, incision infection or empyema, loss of consciousness after surgery or need for mechanical ventilation after surgery, re-operation due to hemorrhage or other thoracic emergencies and patients discharging with chest tube or Heimlich valve.

Using a computer generated randomization provided by a statistician, a surgery resident, not participating in the study, assigned all patients randomly to either the intracostal suture (ICS) group or the intercostal muscle flap and intracostal sutures (ICM) group. A thoracic epidural catheter was placed before the operation. A standard posterolateral thoracotomy with division of the latissimus dorsi muscle and preservation of the serratus anterior muscle was performed by the same surgeon. The pleural space was entered through the fifth or the sixth intercostal space. The cautery was used to open the intercostal space over the upper border of the lower rib.

In the ICM group, a 10 cm to 15 cm segment of the intercostal muscle underneath the upper rib was partially dangled by low energy electrocautery. The chest retractor was put behind the intercostal muscle to avoid crushing it between the lower border of the rib and the upper blade of the retractor. To avoid rib fracture, we attempted to use a piece of gauze between the blade and the rib and slowly opened the chest retractor. When the procedure was completed, patients had one or two 28F soft chest tubes placed.

**Fig. 1:**
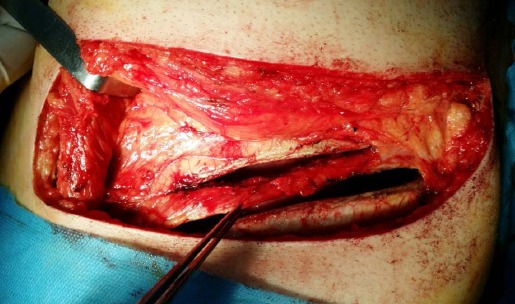
The intercostal muscle underneath the upper rib partially dissected along with the intercostal neurovascular bundle to protect the intercostal nerve above the incision

**Fig. 2:**
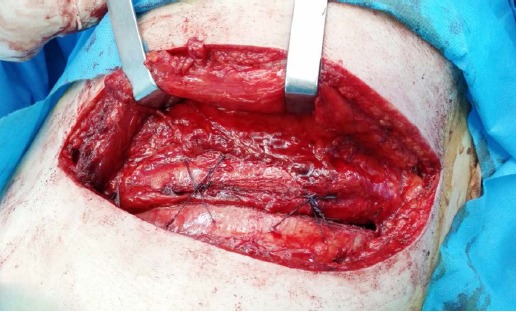
For closure, two intracostal sutures were placed through predrilled holes on the lower rib and over the top of the upper rib.

For closure, three or four intracostal sutures were passed through predrilled holes on the lower rib and passed over the top of the upper rib, according to Cerfolio et al.^9^ (Fig.1 and Fig. 2). In the ICS group, intracostal sutures were used for all closures by passing three to four sutures in holes made by drilling the lower rib and passing over the upper border of the rib corresponding to the space.

**Fig. 3:**
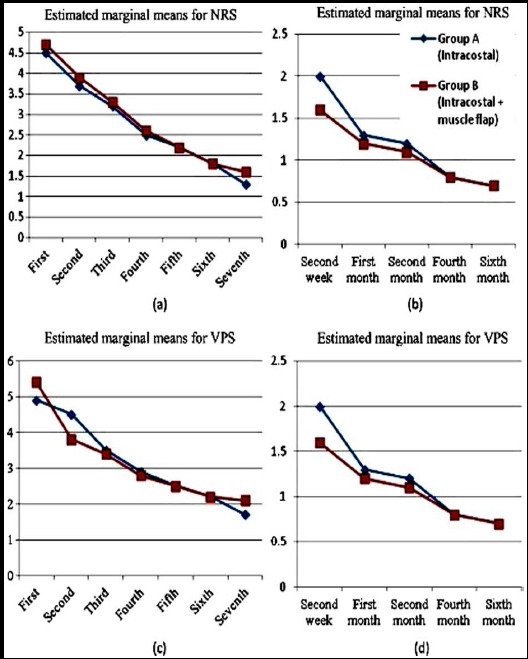
(a) Pain decreasing trend in numeric rating scale (NRS) during hospitalization days (early post-operative pain) (b) Pain decreasing trend in numeric rating scale (NRS) during follow-up visits (late postoperative pain) (c) pain decreasing trend in visual pain scale (VPS) during hospitalization days (early post-operative pain) (d) Pain decreasing trend in visual pain scale (VPS) during follow-up visits (late post-operative pain).

Pain evaluation was performed using two methods of numeric rating scale (NRS) and visual pain scale (VPS). In NRS, zero score means no pain and 10 score means the most severe pain that patients experience. Visual pain score is shown with even digits ranging from 0-10 and is determined by using some standard. Pain evaluation was done in days: 1, 2, 3, 4, 5, 6, and 7 of the hospitalization. After patients’ discharge, the pain evaluation scores were recorded during interviews at post-operative second week and the 1^st^, 2^nd^, 4^th^ and 6^th^ months later (Fig.3). All members of pain service including nurses placing the epidural catheter and those collecting the data, were blinded in choosing patients for each group.

All collected data was analyzed with SPSS 22. Descriptive statistical methods (frequency, percentage, mean, and Standard deviation), chi-square test, independent and paired sample t-test and ANOVA with repeated measures were used to analyze the data. The p value for differences was considered less than 0.05.

## RESULTS

**Fig. 4:**
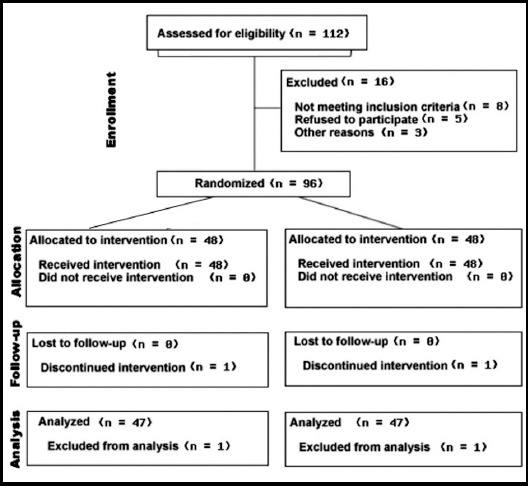
Flow diagram of patients included in the study.

During a 3-year period, out of 112 patients who received posterolateral thoracotomy for various reasons, 96 patients were eligible to participate in the study (Fig.4). No statistical differences were found between the two groups in terms of demographic characteristics (Table-I).

**Table-I:** Demographic characteristics of patients.

	ICS Group	ICM Group	P value
Number of cases	47	47	1.00
Male/Female ratio	1.61	1.61	1.00
Age	45.3 ± 17.6	47.4 ± 16.1	0.36
Mean operation time (min)	191.0 ± 74.7	219.3 ± 68.8	039
Mean admission length (day)	2.5 ± 0.8	2.6 ± 1.0	0.92
Mean time to return to baseline activity	4.6 ± 0.8	5.8 ± 6.6	0.38
Thoracotomy site (Left/Right)	22/24	18/29	0.73

The mean duration for flap preparation in ICM group was 4.5±1.7 minutes. The mean duration of hospitalization for ICS group was 7.0±2.0 days and for group B was 8.9±6.1 days (p>0.05). In ICS group, 33 patients had 5^th^ intercostal incisions, 10 patients had 6^th^ and 4 patients had 7^th^ intercostal incisions to access the thoracic cavity. In ICM group, 4 patients had 4^th^ intercostal incisions, 40 patients had 5^th^ and 3 patients had 7^th^ intercostal incisions. The differences were not statistically significant (p>0.05).

The closure of ICS group in three patients was with two sutures and in 44 patients with three sutures. In ICM group, the closure was with two sutures for five patients and with three sutures for 42 patients. There was no statistically significant difference between them (*P*> 0.05).

Nine patients and eleven patients had one chest tube in ICS group and ICM group, respectively which had not significant difference between two groups (*P*: 0.401). In addition, none of the patients had needed second chest tube, postoperatively.

Regarding intraoperative actions, surgeon tried to reduce rib fracture as much as possible. However, one patient (2.1%) in ICS group and ICM group had one rib fracture. Although, no significant difference was observed between groups in terms of rib fracture (*P*: 0.753); in order to prevent rib fracture effect on patients’ postoperative pain, both patients were excluded from study group.

In terms of postoperative complications due to muscle flap technique, three patients had air leak (all in ICS group), following by atelectasis in 7 patients (six patients in ICS group and one patient in ICM group). (*P*: 0.121). Statistical analysis using chi-square test showed, significant difference observed in terms of atelectasis incidence between groups (*P*: 0.029), Use of epidural catheter, intercostal block and analgesic were obligatory for all patients. Analgesic protocol was based on intravenous paracetamol and morphin. Collected NRS scores and visual scores were regrouped into two time periods, namely as early post-operative pain from day 1 to day 7 and late pain including 2^nd^ week, 1^st^, 2^nd^, 4^th^ and 6 months after thoracotomy (Table-II). There was no statistically significant difference in the mean post-operative pain during hospitalization days and follow-up visits between both groups (p>0.05).

**Table-II:** Pain intensity in numeric scale (NRS), visual pain scale (VPS) during hospitalization days (early, post-operative pain) and follow-up visits (late post-operative pain) between two groups.

		Intercostal suture		Intercostal suture + muscle flap		P value	

		NRS	VPS	NRS	VPS	NRS	VPS
Early post-operative pain (day)	First	4.5 ± 1.5	4.9 ± 1.9	4.7 ± 0.9	5.4 ± 1.5	0.66	0.36
	Second	3.7 ± 1.7	4.5 ± 1.8	3.9 ± 1.1	3.8 ± 1.6	0.80	0.20
	Third	3.2 ± 1.5	3.5 ± 1.6	3.3 ± 1.4	3.4 ± 1.5	0.84	0.92
	Forth	2.5 ± 1.6	2.9 ± 1.8	2.6 ± 1.3	2.8 ± 1.0	0.87	0.92
	Fifth	2.2 ± 1.4	2.5 ± 1.5	2.2 ± 1.2	2.5 ± 1.3	0.98	0.95
	Sixth	1.8 ± 1.0	2.2 ± 1.4	1.8 ± 1.0	2.2 ± 1.2	0.90	0.92
	Seventh	1.3 ± 0.7	1.7 ± 1.2	1.6 ± 0.8	2.1 ± 1.3	0.35	0.34
Late post-operative pain	2nd week	2.0 ± 0.9	2.3 ± 1.2	1.6 ± 0.8	2.4 ± 1.2	0.19	0.79
	1st month	1.3 ± 0.8	1.6 ± 1.0	1.2 ± 0.7	2.1 ± 1.4	0.67	0.26
	2nd month	1.2 ± 0.7	1.1 ± 1.0	1.1 ± 0.9	1.4 ± 1.3	0.70	0.51
	4th month	0.8 ± 0.6	1.1 ± 1.0	0.8 ± 0.8	0.9 ± 1.0	0.82	0.44
	6th month	0.7 ± 0.6	4.9 ± 1.9	0.7 ± 0.7	0.7 ± 0.9	1.00	0.42

The analysis showed that pain decreased during hospitalization and follow up (p<0.001) in both groups. However, the decreasing trend between these two groups was not statistically significant (p>0.001).

## DISCUSSION

Results of the current randomized controlled trial revealed that muscle flap preparation was not significantly effective in post thoracotomy pain improvement, and post-operative pain in intracostal thoracotomy closure was similar to combination of intracostal and ICM flap technique.

Neuropathic pain accounts for most of the post thoracotomy pains.^11^-^13^ Steegers et al. showed that approximately half of the chronic pain after thoracic surgery was not associated with a neuropathic component. More extensive surgery and pleurectomy are predictive factors for chronic pain after thoracic surgery, suggesting a visceral component apart from nerve injury.^12^ However, Maguine et al. demonstrated that thoracotomy with rib resection resulted in more detectable nerve damage than diathermy along the top of the rib and pericostal closure. In addition, they showed that the amount of intra-operative intercostal nerve damage is not indicative of long-term nerve damage or that there is a more significant cause for chronic pain other than intercostal nerve injury.^14^

Different methods are being used for intercostal nerve preservation. Generally, intracostal methods are preferred in comparison to pericostal methods.^8^ Bayram et al. showed significant decrease in post-thoracotomy pain subsequent to thoracotomy closure performed by avoiding intercostal nerve compression after partial dissection of the intercostal muscles. In addition, they indicated that post-thoracotomy pain was much less in patients with rib approximation by the use of intracostal sutures after intercostal nerve dissection.^15^ Yan et al. indicated that protection of bilateral intercostal nerves around the incision contributed to significant pain relief after operation without the increase of the morbidity of complications.^16^

The efficacy of using intercostal muscle flap in diminishing pain is still controversial.^10^ In the current study, intracostal closure was used for both groups while intercostal muscle flap was used for just one group. In a study by Cerfolio et al., intercostal muscle flap role in pain control has been suggested. However, while intercostal muscle flap harvesting without cutting from rib edge added to intercostal closure, it led to pain reduction on postoperative weeks.^9^ Allama et al. evaluated ICM flap role in reduction of post thoracotomy pain, and results showed that ICM flap combined with intracostal sutures provides rapid, safe and much effective technique, compared to other techniques.^17^ On the other hand, Gracia et al. showed that the long term efficacy of intercostal muscle flap in post thoracotomy pain improvement was much more significant.^18^ However, Wu et al. showed that a combination of intracostal suture with intercostal muscle flap may not necessarily lead to better post-thoracotomy pain control than using intercostal suture alone.^19^ There was a same concern during the present trial, whereas using intercostal muscle flap did not have considerable efficacy in pain management after thoracotomy during hospitalization and follow-up periods. Furthermore, despite prevalent neuropathic pattern in most of post thoracotomy pains, it seemed that intercostal nerve preservation with subsequent preparation of intercostal muscle flap did not lead to post-operative pain control.

Potential invisible damage to the intercostal nerve while raising a muscle flap might be a major concern. It seems that pain could be affected by other factors such as the patients’ psychological status and subjectivity of pain sensitivity. The force of retraction and a broad width of entry space may contribute to the intercostal nerve injury. A narrow width of entry space, short operation time and releasing the retractor periodically would be helpful for pain relief.

Although, early and late post thoracotomy pain is a common condition with high neuropathic region, there was no significant difference in the pain management between two approaches used in the current study. Therefore, more clinical trials with larger population group are suggested for further studies. In addition, comparison of posterolateral and anterolateral should be taken into consideration in future studies.

### Limitations

Our study had some limitations. Due to small number of cases referred to our hospital, the study group was small. Hence there is a need for studies with larger population. Secondly, due to lack of patients’ cooperation, we were unable to prolong our follow up period.

## CONCLUSION

Thoracotomy closure with a combination of intracostal sutures and intercostal muscle flap method have more accurate intercostal nerve preservation, has no significant advantage compared with simple intra costal sutures in post thoracotomy pain management.
